# Survey data on the attitudes of adolescents in Hong Kong towards the COVID-19 vaccination

**DOI:** 10.1016/j.dib.2022.108069

**Published:** 2022-03-18

**Authors:** Wilfred Hing-Sang Wong, Daniel Leung, Gilbert T. Chua, Jaime Sou Da Rosa Duque, Sinéad Peare, Hung Kwan So, Sau Man Chan, Mike Yat-Wah Kwan, Patrick Ip, Yu Lung Lau

**Affiliations:** a Department of Paediatrics and Adolescent Medicine, Queen Mary Hospital, Li Ka Shing Faculty of Medicine, University of Hong Kong, P.R. China; b Department of Paediatrics and Adolescent Medicine, Princess Margaret Hospital, Hong Kong, P.R. China

**Keywords:** COVID-19, Vaccine, Public health, Paediatrics, Attitudes

## Abstract

To our best knowledge, this article presents a novel data set on Hong Kong's adolescents’ attitude towards the COVID-19 vaccination, excluding their parental opinions. This research used a cross-sectional questionnaire survey, which collects data from the population at a single point in time. Our questionnaire was designed in both English and Chinese for the adolescents’ convenience, using a self-designed, online questionnaire website, which was sent to 30 secondary schools across Hong Kong at the beginning of June 2021, to be completed by 31st June 2021. This gathered a total of 2609 surveys, excluding those which did not fit into the criteria. As the data has identified factors that affect vaccine hesitancy, government authorities can use the data to choose effective ways to promote the COVID-19 vaccination and to educate the general population about the benefits of receiving it.

## Specifications Table

**Table:** 

Subject	Infectious Diseases
Specific subject area	Attitude of receiving COVID-19 vaccine in adolescents
Type of data	Table
How the data were acquired	Data was collected through an online questionnaire. The questionnaire is provided as supplementary material.
Data format	Raw Analysed
Description of data collection	Data was collected through an online questionnaire, using the link: covayouthwill.hkuhealth.com, which was self-developed by the Department of Paediatrics and Adolescent Medicine, Queen Mary Hospital, Li Ka Shing Faculty of Medicine, University of Hong Kong. The questionnaire was shared to 30 secondary schools across Hong Kong, who have participated in previous vaccination-related research conducted by the Department of Paediatrics and Adolescent Medicine.
Data source location	• Institution: Department of Paediatrics and Adolescent Medicine, Queen Mary Hospital, Li Ka Shing Faculty of Medicine, University of Hong Kong • Country: Hong Kong
Data accessibility	The raw data “Survey data on the attitudes of adolescents in Hong Kong towards the COVID-19 vaccination” linked to the tables can be found from the data repository at https://data.mendeley.com/datasets/7gc9mxbb3b/2
Related research article	Wong WHS, Leung D, Chua GT, Duque JSR, Peare S, So HK, Chan SM, Kwan MYW, Ip P, Lau YL. Adolescents' attitudes to the COVID-19 vaccination. Vaccine. 12 (2022) S0264–410X(22)00,025–1. doi: 10.1016/j.vaccine.2022.01.010[1].

## Value of the Data

• This data provides information on what factors and reasons affect vaccine hesitancy and willingness in adolescents in Hong Kong, which is important for understanding how to improve the general uptake of the COVID-19 vaccine.
• Although parental consent is required for adolescents to be vaccinated in Hong Kong, this data gives an understanding of adolescents’ thinking and the associating factors for their vaccination behaviours.
• The data can be useful for researchers who want to further investigate the factors and reasons for vaccine acceptance/hesitancy in adolescents.
• The dataset can be used to further research and understand how the COVID-19 vaccine is discussed and viewed among the adolescents. A comparison to a future study can be done to see the change in attitudes and opinions after the implementation of interventions.
• The data can assist the Hong Kong Government and other national governments in choosing effective ways to promote the COVID-19 vaccination and to educate the general population about the benefits of receiving it.

## 1 Data Description

The survey data of the attitude of receiving COVID-19 vaccine in adolescents from June 7, 2022 to June 30, 2022 were collected from the official website (covayouthwill.hkuhealth.com) of online questionnaire which was self-developed by the Department of Paediatrics and Adolescent Medicine, Queen Mary Hospital, Li Ka Shing Faculty of Medicine, University of Hong Kong. Tables 1 and 2 show the top ten reasons that associate with intention to receive COVID-19 vaccination or not among the participants.

**Table 1:** Top ten reasons of plan to receive the COVID-19 vaccine.

Reasons	Ranking
I am worried that I will be infected	1
I want to protect my family	2
I wish to return to my life before COVID-19 pandemic	3
I want to travel	4
I want to play sports at school with friends or in a competition without wearing mask	5
I want to participate in large-scale events (e.g. open days, joint school events and Christmas balls) without wearing mask	6
Socialize with large groups of friends, (e.g. dining out, karaoke, party room)	7
I want to resume full scale face-to-face teaching.	8
I am tired of the social distancing policies	9
I want to study overseas	10

**Table 2:** Top ten reasons of not plan to receive the COVID-19 vaccine.

Reasons	Ranking
I am concerned about its safety	1
I am concerned about its efficacy	2
Facemask and social distancing are sufficient	3
I am against vaccination	4
Depends on the progress of the pandemic	5
Not allowed by my parents	6
I do not know whether I should receive the vaccine	7
Medically not suitable (e.g. severe allergies, uncontrolled diabetes mellitus)	8
I do not know which type of COVID-19 vaccine I should receive	9
COVID-19 is just a mild disease	10

The questionnaire is provided as a supplementary file. Supplementary material shows the online questionnaire that all the students had to complete. It contains a series of yes-no questions and multiple-choice questions. The multiple-choice questions have an “Others (please specify)” option. The questionnaire is split into two sections: “Parental information” and “Student's information”, with the latter being the main section.

## 2 Experimental Design, Materials and Methods

Vaccine hesitancy remains a significant barrier to achieve herd immunity [2]. The WHO has even considered it to be one of the leading threats to public health in 2019 [3]. Vaccine hesitancy refers to the delay in acceptance or refusal of vaccination despite the availability of vaccination services [4], [5]. It is influenced by factors classified into three main categories: confidence (lack of trust in the safety and efficacy of a vaccine), complacency (low perception of disease risk) and convenience (availability and affordability) [4], [5]. The factors and reasons listed in the questionnaire (supplementary material) are inspired by these three categories.

The dataset was collected using a cross-sectional questionnaire survey. 33 secondary schools from 637 secondary schools in Hong Kong were invited randomly to answer the questionnaire, but 3 schools withdrew due to a similar study being conducted by the Hong Kong Government at a similar time. The questionnaire was shared with 30 secondary schools across Hong Kong, who have participated in previous vaccination-related research or have been involved in education seminars conducted by the Department of Paediatrics and Adolescent Medicine, the University of Hong Kong. The 30 secondary schools covered 14 out of the 18 districts in Hong Kong. In total 3662 participants from 3 to 4 classes selected by the participated schools were invited to join this survey. The survey ran for a month – it was sent at the beginning of June 2021 and was required to be completed by 30th June 2021. A total of 2609 surveys were collected, after excluding 224 participants that were not within the adolescent age range (12 to 18 years) and 82 due to incomplete answers.

The data was downloaded as an Excel spreadsheet from the self-developed online questionnaire website (covayouthwill.hkuhealth.com). The data was then imported into the digital system IBM SPSS Statistic 21. The top ten reasons of intend to receive COVID-19 vaccine is listed in Table 1. The top ten reasons of not intend to receive COVID-19 vaccine is listed in Table 2.

## Ethics Statements

This study has been approved by the Hong Kong University/Hospital Authority Hong Kong West Cluster IRB Committee (IRB No: UW 21–157). Informed consent was collected from all those participating. The legal guardians of all participants have agreed with the participation of the minors in the study.

## CRediT authorship contribution statement

**Wilfred Hing-Sang Wong:** Formal analysis, Methodology, Resources. **Daniel Leung:** Software. **Gilbert T. Chua:** Methodology, Writing – original draft. **Jaime Sou Da Rosa Duque:** Methodology, Writing – original draft. **Sinéad Peare:** Formal analysis, Writing – original draft. **Hung Kwan So:** Resources. **Sau Man Chan:** Investigation. **Mike Yat-Wah Kwan:** Resources. **Patrick Ip:** Resources. **Yu Lung Lau:** Conceptualization, Methodology, Writing – review & editing.

## Declaration of Competing Interest

The authors declare that they have no known competing financial interests or personal relationships that could have appeared to influence the work reported in this paper.

## Data Availability

Survey data on the attitudes of adolescents in Hong Kong towards the COVID-19 vaccination (Original data) (Mendeley Data).

## Appendix Supplementary materials

Image, application 1
