# Supplementary material for: Survey data on the attitudes of adolescents in Hong Kong towards the COVID-19 vaccination
Source: Data Brief. 2022 Mar 18;42:108069. doi: 10.1016/j.dib.2022.108069 (PMC8932007; doi:10.1016/j.dib.2022.108069)
Supplement: Supplementary file 1 [file mmc1.pdf]

Willingness to receive a COVID-19 vaccine among secondary school students in Hong Kong

Online questionnaire

Parental information

Did your parents receive COVID-19 vaccine?

Father ☐Yes ☐No

Mother ☐Yes ☐No

Did any OTHER family member receive COVID-19 vaccine?

☐Yes ☐No

Student's information

Age (years): ________

Gender

☐ Male

☐Female

Did you get the flu vaccine last year?

☐Yes

☐No

Have you received the flu vaccine every year in the past three years?

☐Yes

☐No

Have you been diagnosed with COVID-19?

☐Yes

☐No

Have your family members or classmates been diagnosed with COVID-19 disease?

☐Yes

☐No

Have you ever been classified as a close contact of COVID-19 that needs to undergo compulsory quarantine?

☐Yes

☐No

Have you ever been required to compulsorily test the COVID-19 virus?

☐Yes

☐No

Have you or do you plan to receive the COVID-19 vaccine if you were eligible?

☐Yes

Because: (you can choose more than one choice)

☐I am worried that I will be infected

☐I want to protect my family

☐I want to attend religious ceremonies

☐I want to travel

☐I wish to return to my life before COVID-19 pandemic

☐I am tired of the social distancing policies

☐Because people around me received vaccination

☐I want to resume full scale face-to-face teaching.

☐I want to play sports at school with friends or in a competition without wearing mask

☐I want to play music safely (e.g. wind instruments, band, choir)

☐I want to participate in large-scale events (e.g. open days, joint school events and Christmas balls) without wearing mask
☐I want to study overseas
☐I want to join study tours
☐Socialize with large groups of friends, (e.g. dining out, karaoke, party room)
☐Others (please specify):________

☐No, because (choose the most important reason)

☐Not allowed by my parents

☐Medically not suitable (e.g. severe allergies, uncontrolled diabetes mellitus)

☐I am concerned about its safety

☐I am concerned about its efficacy

☐I am concerned that receiving the vaccine will violate the rules of my religion

☐COVID-19 is just a mild disease

☐I do not know where to receive the vaccines

☐I do not know whether I should receive the vaccine

☐The community vaccine centres are inconvenient

☐I do not know which type of COVID-19 vaccine I should receive

☐Facemask and social distancing are sufficient

☐Depends on the progress of the pandemic

☐I am against vaccination

☐Others (please specify):________

Will you continue to receive the COVID-19 vaccine if it has to be administered annually?

☐Yes

☐I prefer to receive the same type of vaccine that I received previously

☐I prefer to receive a different kind of the COVID-19 vaccine

☐No

香港中學生接種新冠疫苗的意願調查

網上問卷

家長信息部份

您的父母是否接種了COVID-19疫苗？

父親　☐是　☐否

母親　☐是　☐否

有其他家庭成員接種過COVID-19疫苗嗎？

☐有　☐沒有

學生資料

年齡（歲）：________

性別

☐男性

☐女性

請問你去年有沒有接種流感疫苗？

☐有

☐沒有

請問你在過往三年有沒有每年接種流感疫苗？

☐有

☐沒有

您曾被診斷出患有COVID-19嗎？

☐是

☐否

請問你的家人或同學有沒有曾經被確診患上COVID-19？

☐有

☐沒有

請問你有沒有曾經被界定為COVID-19的密切接觸者而需要接受強制隔離？

☐有

☐沒有

請問你有沒有曾經被要求強制檢驗COVID-19？

☐有

☐沒有

如果您符合條件，您是否打算接受COVID-19疫苗？

☐是

因為：（您可以選擇多於一個原因）

☐我擔心我會被感染

☐我想保護我的家人

☐我想參加宗教儀式 (例如：崇拜)

☐我想去旅行

☐我希望重返在COVID-19大流行之前的生活

☐我對社交距離政策感到厭倦

☐因為我周圍的人都接種了疫苗

☐我想全面恢復面授教學

☐我想在學校和朋友一起參加體育運動或參加比賽而不戴口罩

☐我想安全地參與音樂活動（例如：管樂器, 樂隊, 合唱團)

☐我想不戴口罩參加大型活動（例如：開放日、聯校活動和聖誕舞會）

☐我想出國留學

☐我想參加遊學

☐可以與一大群朋友聚會，(例如外出用餐、卡拉OK、派對室)

☐其他 (請註明）：________

☐否，因為（您可以選擇多於一個原因）

☐我的父母不允許

☐身體不適合接種（例如嚴重過敏，不受控制的糖尿病）

☐我擔心它的安全性

☐我擔心它的功效

☐我擔心接種疫苗會違反我的宗教信仰

☐COVID-19只是一種輕度疾病

☐我不知道在哪裡接種疫苗

☐我不知道我是否應該接種疫苗

☐社區疫苗中心不方便

☐我不知道我應該接受哪種類型的COVID-19疫苗

☐口罩和社交距離就足夠了

☐取決於大流行的進展

☐我反對接種疫苗

☐其他（請註明）：________

如果必須每年接種一次，您會繼續接種 COVID-19 疫苗嗎？

☐ 會

☐我希望接受與以前相同類型的疫苗

☐ 我更願意接受不同種類的 COVID-19 疫苗

☐ 否
